# Strengthening management and leadership practices to increase health-service delivery in Kenya: an evidence-based approach

**DOI:** 10.1186/1478-4491-10-25

**Published:** 2012-08-29

**Authors:** La Rue K Seims, Juan Carlos Alegre, Lily Murei, Joan Bragar, Nandita Thatte, Peter Kibunga, Sammuel Cheburet

**Affiliations:** 1 Management Sciences for Health, 4301 North Fairfax Drive, Suite 400, Arlington, VA, 22203, USA; 2 Management Sciences for Health, Kenya (former), Box 61379, Nairobi, 00200, Kenya; 3 Management Sciences for Health, 784 Memorial Drive, Cambridge, MA, 02139, USA; 4 Public Health Institute Global Health Fellow, US Agency for International Development, Office of Population and Reproductive Health, United States Agency for International Development, 1300 Pennsylvania Avenue, Washington, DC, 20523, USA; 5 Management Sciences for Health, ACK Garden House, Block A, 6th Floor, First Ngong Road Avenue, off Bishops Road, PO Box 8700-00100, Nairobi, Kenya; 6 Ministry of Health, Afya House, Cathedral Road, PO Box: 30016-00100, Nairobi, Kenya

**Keywords:** Health worker performance, Institutional strengthening, Kenya, Leadership, Management, Service delivery

## Abstract

**Background:**

The purpose of the study was to test the hypothesis that strengthening health systems, through improved leadership and management skills of health teams, can contribute to an increase in health-service delivery outcomes. The study was conducted in six provinces in the Republic of Kenya.

**Methods:**

The study used a non-randomized design comparing measures of key service delivery indicators addressed by health teams receiving leadership and management training (the intervention) against measures in comparison areas not receiving the intervention. Measurements were taken at three time periods: baseline, endline, and approximately six months post intervention. At the district level, health-service coverage was computed. At the facility level, the percentage change in the number of client visits was computed. The t-test was used to test for significance.

**Results:**

Results showed significant increases in health-service coverage at the district level (p = <0.05) in the intervention teams compared to the comparison teams. Similarly, there were significant increases in the number of client visits at the facility level in the intervention group versus comparison facilities (*P* < 0.05).

**Conclusions:**

Strengthening the leadership and management skills of health teams, through team-based approaches focused on selected challenges, contributed to improved health service delivery outcomes and these improvements were sustained at least for six months.

## Background

 In recent years there has been an increased interest in health systems strengthening and building capacity in developing countries to ensure the sustainability of public health programs. The World Health Organization (WHO) has defined a health system as one which includes the organizations, institutions, resources, and people whose primary purpose is to improve health. WHO has also identified six critical building blocks for a well-functioning health system: service delivery, human resources for health, medical products, health information systems, health financing, and leadership and governance [1]. The final building block, leadership and governance, is one of the most challenging to measure and thus the one for which there is little empirical evidence for its impact on service delivery and health outcomes.

 Changes in health systems strengthening efforts, especially through leadership training and team training approaches, have been associated with changes in service delivery in health-care settings or in health outcomes in only a few peer-reviewed studies [2,3]. Most studies assessing leadership in medical or public health settings focus on provider skill acquisition rather than service delivery or health outcomes [4,5]. Review of the grey literature around teamwork practices and patient safety further supports the conclusion that “only limited evidence exists regarding which process and outcome measures are most directly and strongly affected by changes in teamwork effectiveness” [6].

A previous study of the Leadership Development Program (LDP) intervention, also used in this study, was conducted by Topçuoğlu in Egypt using a before-after design, without a comparison group. In 2003, after participating in the LDP, 10 teams from three districts increased the number of new family planning visits by 36%, 68% and 20%, respectively and the approach was later scaled up to 184 facilities. This increase was associated with a reduction in the maternal mortality rate, the goal addressed by the LDP teams, from 85.0 per 100 000 live births to 35.5 per 100 000 in Aswan Governorate [7]. The study also found that, even though only new family planning visits were being targeted, prenatal care and postnatal care visits also increased.

In this study, the hypothesis tested was that strengthening health systems, especially leadership and management skills of health teams, will result in increased health services relative to a comparison group.

The study was implemented in districts and facilities in six provinces in the Republic of Kenya. At the district level, public health services are managed by the District Health Management Team (DHMT) and the Public Health Unit of district hospitals. The DHMT and District Health Management Board provide management and supervision support to rural health facilities, including subdistrict hospitals, health centres, and dispensaries. District and subdistrict hospitals deliver health-care services, including inpatient and outpatient services. Health centres concentrate on ambulatory services and preventive services. Dispensaries are intended to be the first line of contact with patients and provide a large range of preventive services and primary care.

## Methods

### Study design and matching

The study used a quasi-experimental design with comparison groups but without random assignment. The study compared outcomes of teams that participated in a LDP intervention against comparison groups that did not participate in the intervention. Outcomes included measures of a key indicator addressed by each of 67 teams. Measurements of health-service indicators were taken from both intervention and comparison groups at three time periods: before the LDP (baseline), six months later at the end of the LDP (endline), and approximately six months after the LDP ended to assess if the results were sustained (post intervention). All 67 teams had quantifiable indicators measured in the Government of Kenya Health Management Information System (HMIS) for which comparison data could be collected [8]. Data on changes in service delivery for intervention teams were compared with data on changes in the same indicators in the HMIS in comparison areas.

 Teams participating in the intervention were included from the Rift Valley, Nyanza, Central, Eastern, North Eastern, and Nairobi Provinces in Kenya. Teams were self-selected based upon interest in participating in the LDP intervention. In the absence of random assignment, intervention districts and facilities were matched with comparison districts and facilities not receiving the intervention. Matching was done by a team of independent consultants at Harvard University School of Public Health, Department of Population and International Health. Districts and facilities were matched separately using the Coarsened Exact Matching program in Stata v. 11. This method allows for matching on multiple criteria and for matching to more than one district or facility [9].

At the district level, 16 districts received the intervention, representing 18 district-level teams. A total of 58 districts which had not received the LDP intervention were identified as possible matches. Districts were not matched one-to-one. Rather, the set of districts that addressed a specific indicator were matched with a set of districts that were similar with regard to geographic location and district population. The matched comparison districts were in Rift Valley, Nairobi, and Nyanza Provinces. Teams had previously been trained in the LDP method from all districts in Eastern, Central, Coast, and North Eastern Provinces, rendering them unavailable to serve as comparison areas at the district level.

At the facility level, facilities were also not matched one-to-one. Criteria for selecting matches also included geography, with facilities in northern areas matched with northern areas, facilities in central areas matched with central areas, and facilities in southern areas matched with southern areas. Additional matching criteria were type of facility, using categories established by the government HMIS (i.e. “dispensary, health centre, primary hospital, other hospital, and other private hospital”), number of beds, and family planning service volume. A total of 1254 potential comparison facilities from the Rift Valley and Nyanza Provinces were used in identifying the closest matches.

Table 1 shows the number of district level and facility teams by measureable result in both intervention teams and matched comparisons.

**Table 1:** Number of intervention and comparison districts and facilities by indicator, Kenya programmatic assessment, 2009 to 2010

**Indicator**	**Number of intervention districts**	**Number of comparison districts**
Fully-immunized child	11	10
Deliveries by skilled birth attendant	3	3
Others	4	2
**Indicator**	**Number of intervention facilities**	**Number of comparison facilities**
Fully-immunized child	14	12
Deliveries by skilled birth attendant	20	17
Four or more antenatal care visits	10	10
Others	5	5
Total	67	59

### Intervention

 The LDP intervention is an approximately six-month program that uses a team-based approach to develop leadership and management skills among health workers. A total of 13 of the 67 teams participated in the program in 2008, 53 in 2009 and 1 in 2010. The intervention centres around a “Challenge Model” whereby participants select a problem or challenge faced and develop a shared vision and action plan to help address the challenge as a team [10]. Additional components include: *stakeholder alignment* meetings at the national and subnational levels to generate commitment to and ownership of the LDP among decision makers; *four LDP workshops* that train participants in various leadership practices including scanning, focusing, aligning and mobilizing, and inspiring. *on-the-job team meetings* where teams work on action plans to address the selected challenge and plans for monitoring progress in achieving measurable results; and *meetings with mentors/coaches* where teams review and reinforce LDP content and receive technical assistance for monitoring and evaluating progress on their action plans.

**Figure 1:**
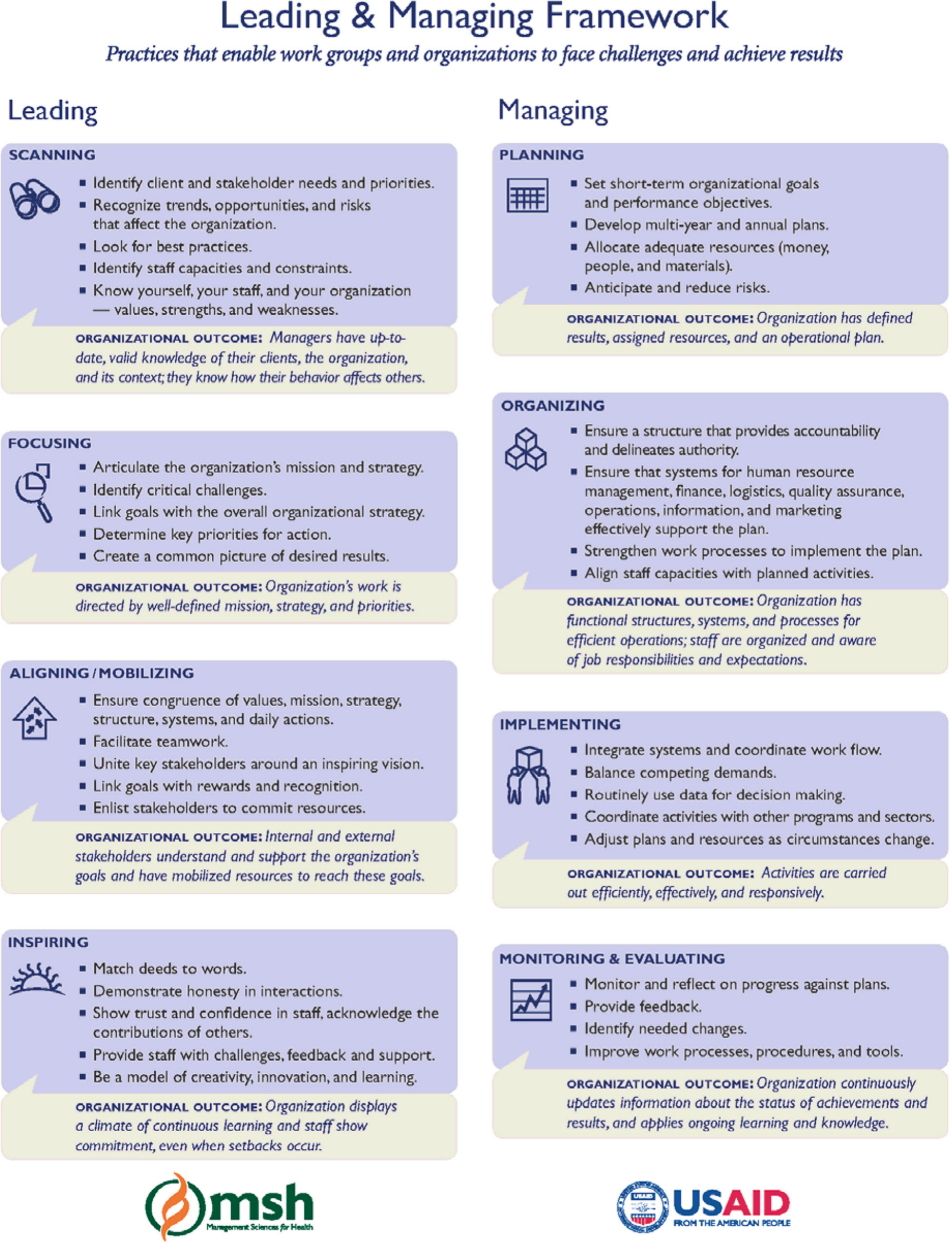
**Leading and Managing Framework.** Practices that enable work groups to face challenges and achieve results.

The Leading and Managing Framework, which guides the LDP intervention, is included in Figure 1.

Adapted from several models, including Kotter’s leading and managing practices [11], the framework shows a process that enables managers and their teams to “face challenges and achieve results”. This definition draws on the work of Heifetz [12], which sees leading not as a matter of personality or charisma, but as a function that can be done at any level, turning the work back to those who need to learn and adapt to face challenges and achieve progress.

### Respondents

A total of 67 intervention teams of health managers, doctors and nurses were included in the study conducted in 2009 to 2010 in collaboration with the Kenya Ministry of Health. Data were collected from team leaders either in-person or during telephone interviews. Eighteen teams implemented health interventions that covered all or most of their respective districts, and 49 teams implemented health interventions in the facilities in which they worked from district hospital to dispensary level. District teams were often comprised of members of the DHMT. The 67 teams included in the analysis addressed increasing coverage of fully-immunized children under one year (25 teams), delivery by a skilled birth attendant (23 teams), 4 or more antenatal care visits (11 teams), and other health-care challenges (8 teams). The level of team is detailed by intervention in Table 1.

### Data collection and analysis

 Data were collected for the intervention teams from February to April 2010 by contacting each team leader by email and/or telephone to confirm the results reported at the time of each LDP and to obtain additional data on post intervention results and the factors that supported or hindered the sustainability of the results.

Data for comparison areas were collected in August 2010 by Government of Kenya HMIS Officers. The HMIS Officers extracted data from service delivery registers and district health records for the three time periods of the study. Data were available for nearly all comparison districts and facilities and time periods requested, except for male and female outpatient department visits, where data were available from only one comparison facility. Qualitative data were collected during in-person or telephone interviews with each LDP team leader.

Quantitative data were double-checked, entered and analysed using Microsoft Excel. Significance tests were calculated using the Statistical Package for the Social Sciences (SPSS) v. 18.0.0. Qualitative data were entered and coded for content using NVivo v.8.0.340.

The percentage coverage for each of the 67 intervention teams was averaged for each of the three time periods in the analysis—baseline, endline, and post intervention—with each team given the same weight.

The results of each district-level LDP team were then compared with the averaged results for the group of comparison districts to which it was matched for the exact same time periods and indicators. For most comparison facilities, catchment areas had not been established by the Kenya HMIS. Therefore, because the percentage coverage could not be calculated for comparison facilities, the basis of the analysis at the facility level was the percentage change in numbers served from baseline to endline, and from baseline to post intervention. Results, therefore, are presented separately for districts and facilities as the percentage coverage could be computed at the district level but only the numbers served were available at the facility level. Significance tests were calculated using SPSS v. 18.0.0.

## Results and discussion

**Figure 2:**
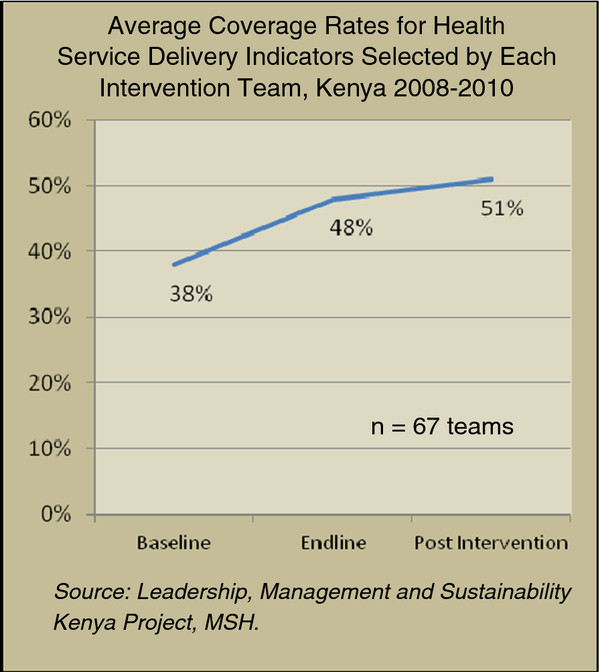
Average coverage rates for health-service delivery indicators selected by each intervention team, Kenya 2008–2010.

The combined results for the 67 LDP teams included in the analysis are shown in Figure 2.

For all 67 teams at district and facility levels that received the LDP intervention, the average coverage rate for selected health-service delivery indicators was 38% at baseline, 48% at endline, and 51% post intervention, showing that the teams, on average, were able to improve their measureable results and to sustain the improvements post intervention, at least for six months. The average coverage for the 67 teams was computed by averaging 67 measures, one measure for each team, of the proportion of the target population covered for an indicator selected by the team for the relevant time period.

**Figure 3:**
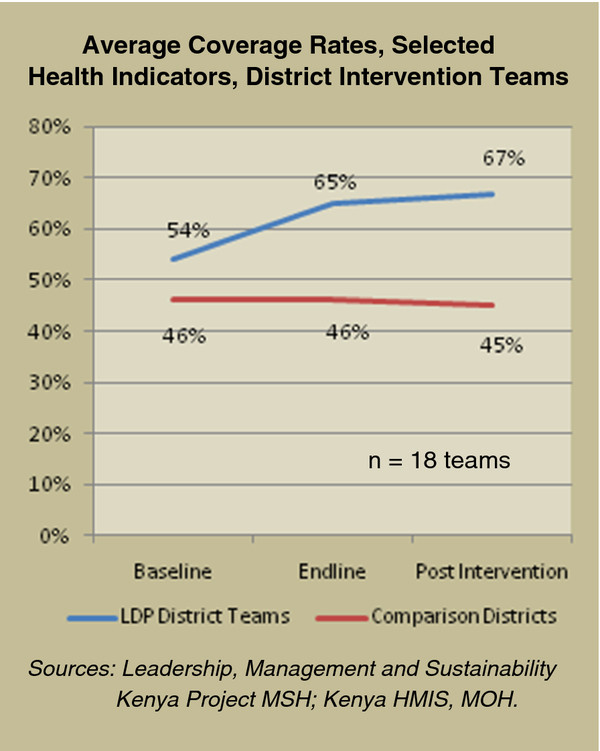
Average coverage rates, selected health indicators, district intervention teams.

From the qualitative interviews with LDP team leaders, we found that for those teams able to sustain coverage after the end of the LDP (about two thirds of the teams), contributing factors were cited to be increased demand generated through social mobilization and health education, increased access by providing more outreach sites or more service hours or days, and an improved work climate due to renovated staff quarters, training, or supervision. For the third of teams unable to sustain results, the major factors cited were staff shortages that led to decreased access (e.g. fewer outreach sites, mobile services suspended, or long lines, especially on market days). Most frequently cited secondary reasons included drought and insecurity and a shortage of medicines or supplies, especially vaccines. Results for district-level teams in relation to comparison districts are shown in Figure 3.

 For the 18 district-level teams that received the LDP intervention, the average coverage rate for selected health indicators increased from 54% at baseline to 65% at endline, and 67% post intervention. Each team at district level selected one health indicator to address. The selected indicators included fully-immunized children under one year old (11 teams), women who delivered with a skilled birth attendant (3 teams), and others (4 teams). Other indicators comprised institutional strengthening indicators that impact upon service delivery and access to services: timely and accurate reporting of service statistics (two teams), proportion of women of reproductive age receiving family planning commodities (one team) and number of rural delivery sites (one team). Comparison areas remained stable, with aggregated coverage rates of 46%, 46%, and 45%, respectively, for the same three time periods. Difference in means from baseline to endline measures for the treatment and comparison groups at the district level were highly significant (*P* = 0.001). The difference in means from baseline to the post intervention measures for the two groups was also significant (*P* = 0.0465, t-test).

There was a discrepancy of eight percentage points (54% compared with 46%) comparing the baseline for intervention districts to the baseline for matching districts. This most likely was due to the limited criteria at district level for which we had data on which to base the matches selected.

At the facility level, the number of clients served more than doubled in areas where the LDP had been implemented, with LDP teams reporting an increase in results of 121% from baseline to endline, and 137% from baseline to post intervention for selected health indicators. Indicators addressed by teams at facility level included fully-immunized children under one year old, women who delivered with a skilled birth attendant, pregnant women who had four or more antenatal care visits, and others. Comparison facilities showed a very modest increase of 9% from baseline to endline and 26% from baseline to post intervention.

Difference in means from baseline to endline measures for the treatment and comparison groups at the facility level were highly significant (*P* = 0.0015; t-test) as were the difference in means from baseline to the post intervention measure for the two groups (*P* = 0.0105).

Despite the collaborative input of the Ministry of Health and other stakeholders, there were challenges and limitations in conducting this study. First, LDP teams selected their own challenge and measurable results on which to focus. Thus, the indicators varied from team to team and the analysis had to focus on average coverage or service volume rather than on specific indicators. The lead authors acknowledge this limitation of the study. While the focus of this study was upon team performance rather than impact upon a particular health area, we recommend that a future study focus on either teams addressing the same indicator or a set of related indicators for greater methodological rigor.

Second, the teams made their own decision to participate in the intervention, resulting in the possibility of selection bias. Matching on multiple criteria limited this threat to validity but did not completely protect against it.

 Third, contamination of comparison sites could have occurred if regional and district level managers who supervise both comparison and intervention sites may inadvertently have transmitted LDP tools and approaches to the comparison sites or if an LDP team member was transferred to a comparison site. A total of 37% of the teams had had at least one team member transferred to a new area at the time of data collection.

## Conclusions

Given the emphasis on health systems strengthening in the global public health community, this study provides new evidence that interventions designed to strengthen leadership and management produce positive changes in health-service delivery outcomes that can be sustained for at least six months post intervention. This type of management and leadership training intervention may be useful to strengthen the health system and improve health outcomes of vulnerable and disadvantaged populations in similar settings in sub-Saharan Africa.

## Abbreviations

DHMT: District Health Management Team; HMIS: Health management information system; LDP: Leadership Development Program; WHO: World Health Organization; SPSS: Statistical Package for the Social Sciences.

## Competing interests

The authors declare that they have no competing interests.

## Authors’ contributions

LS directed the study, entered and analysed the data, and was the major writer of the article; JCA contributed to data review and analysis and writing; JM contributed substantially to developing the intervention, researching the background, and writing sections on the intervention; LM supervised all data collection for the intervention teams, locally managed the study, and contributed to writing the methodology; PK coordinated data collection for comparison areas, entered the data, and contributed to analysis and reporting of comparison data; NT contributed to developing the methodology, defining the larger context, and writing the report; SC supervised all data collection for the comparison areas. All authors reviewed the final manuscript.
